# When Fear Feels Like Fact: Cognitive Bias and Cardiac Risk Perception in Youth Sports

**DOI:** 10.1016/j.jaccas.2026.107010

**Published:** 2026-03-25

**Authors:** Thomas J. Fiedorek, Brian K. Eble, Joshua A. Daily

**Affiliations:** a Arkansas Children's Hospital, Little Rock, Arkansas, USA; b University of Arkansas for Medical Sciences, Little Rock, Arkansas, USA

**Keywords:** pediatric cardiology, sports clearance, sudden cardiac death

## The Moment When Fear Went Viral

In January 2023, the sports world watched in shock as Damar Hamlin, a safety for the Buffalo Bills of the National Football League, collapsed from commotio cordis on national television during Monday Night Football. In the immediate aftermath, our pediatric cardiology clinics experienced a surge of anxious families. Parents of healthy athletes—who had played safely for years—suddenly visualized what they had never imagined: that each game or practice could be their child's last. The epidemiology of sudden cardiac death in athletes did not change that night, but the perception of it did. This Viewpoint article explores the cognitive biases that distort how cardiac risk in sports is understood and offers strategies to help clinicians reframe these fears.

## The True Risk

Sudden cardiac death remains exceptionally uncommon in young athletes, with large cohort studies demonstrating approximately 0.24 to 6.8 deaths per 100,000 athlete-years.^1^ While much attention is focused on rare cardiac risks, sports participation provides substantial protective benefits—not only for cardiovascular health, but also for emotional well-being, identity formation, resilience, social connection, and mental health.^2^^,^^3^ Conversely, inactivity carries equally serious but far more common risks, including obesity, hypertension, depression, and anxiety.^2^^,^^4^^,^^5^

## The Psychology of Perceived Risk

When a young athlete collapses on television, families do not interpret the moment from an epidemiological standpoint, but from a psychological one. Emotion, uncertainty, and vivid imagery activate heuristics—adaptive mental shortcuts shaped to favor survival, respond to threat, and guide action when information is incomplete. However, when faced with a rare, frightening event such as sudden cardiac arrest in an athlete, those same heuristics can misfire. What normally aids efficient thinking becomes distorted, producing cognitive biases that exaggerate rare, dramatic risks while obscuring more common but equally consequential harms, including those related to inactivity, isolation, or mental health. In sports cardiology, these distortions tend to fall into 4 recurring patterns: emotion-driven bias, probability distortion, social amplification, and clinician susceptibility (Table 1).

**Table 1:** Cognitive Biases in Cardiac Risk Perception and Recommended Counseling Approaches

Bias	Definition	How It Distorts Risk Perception	Clinical Consequence	Counseling Approach
Affect heuristic	Using emotional reactions as a substitute for factual reasoning—when something feels dangerous, it is judged as dangerous, regardless of actual risk.	Emotion overrides analysis; if a cardiac event feels terrifying, it seems likely, even when extremely rare.	Fear-based decisions lead to unnecessary testing or sports restriction, despite reassuring data.	Begin with emotional validation, then gradually introduce numbers, context, and visual comparisons.
Ambiguity aversion	Preference for certainty, even when uncertain options may be safer or more appropriate.	Families equate uncertainty with danger and assume more testing must equal more safety.	Cascades of low-yield testing, false positives, and increased anxiety.	Clarify what testing can and cannot change; name uncertainty honestly.
Anticipated regret	Decisions driven by fear of future guilt or self-blame if something goes wrong.	“If something happens and I didn't act, I'll never forgive myself.”	Leads to excessive testing or removing children from sports “just in case.”	Use shared decision-making to weigh benefits and trade-offs, not just worst-case scenarios.
Availability bias	Estimating frequency or likelihood based on how easily examples come to mind.	Vivid, emotional, or recent events feel common, even if extremely rare.	Families overestimate sudden cardiac arrest risk and seek unnecessary evaluation.	Use natural frequencies and relatable comparisons to help families understand actual risk.
Availability cascade	Repetition through media and conversation makes a rare event seem common.	High-profile cases feel widespread simply because they are widely discussed or seen.	Families assume sudden cardiac death is common; clinics see surges in referrals.	Contrast media visibility with true prevalence; emphasize real-world epidemiology.
Base-rate neglect	Tendency to ignore general statistical frequency (base rates) when assessing risk, instead relying on specific or vivid examples.	Inflates perceived risk of rare events while downplaying common harms tied to inactivity, isolation, or depression.	Overestimates cardiac risk and underestimates harms from inactivity, isolation, or depression.	Emphasize base rates and contrast with more common everyday risks.
Commission bias	Tendency to favor action over inaction—often to avoid feeling responsible if something goes wrong—even when inaction may be safer or more appropriate.	Families equate doing more—tests, restrictions, referrals—with being safer, even when it adds no benefit and may create harm.	Overtesting, sports restriction, or excessive referrals.	Validate the urge to act, then reframe the visit as meaningful action: “You've already done the responsible thing by having them evaluated. Based on what we know, the safest and healthiest choice is to let them continue to play.”
Negativity bias	Giving more weight to negative outcomes than positive or protective ones.	Catastrophic risks (death) overshadow benefits (resilience, health, mental well-being).	Families undervalue the protective benefits of sports participation.	Rebalance risk by highlighting the emotional, physical, and social benefits of participation.
Probability neglect	When the mere possibility of a catastrophic outcome feels intolerable, leading people to treat very low-probability risks as if they were likely.	Families admit the risk is “extremely small,” yet still treat any remote possibility as intolerable—treating possibility as probability.	Leads to disproportionate fear of rare cardiac events, pressure for extensive testing or sports restriction, and inability to be reassured by numbers alone.	Acknowledge that rare tragedies feel impossible to ignore, but gently refocus on likelihood: “Yes, it *could* happen—but how *likely* is it, and what risks would we take on by trying to eliminate even tiny possibilities?”
Zero-risk bias	Preference for eliminating risk entirely, even when zero risk is unattainable.	Parents fixate on removing all risk, overlooking that no decision—including inaction—is risk free.	Withdrawal from sports, unnecessary testing, or avoidance of participation.	Clarify that zero risk is unattainable. Help families choose which risks to carry—and which benefits to protect—rather than trying to eliminate risk entirely.

### Emotion-driven distortions: When fear feels like fact

Highly emotional and memorable events—like a televised cardiac arrest—feel more common than they are. The availability and affect heuristics amplify emotional reactions while suppressing statistical reasoning. In this context, families are not asking, “How likely is this?” but rather, “How could I live with myself if I didn't do everything possible to prevent it?”

### Probability-based distortion: When possibility overwhelms probability

Once an event becomes personally imaginable, we often substitute vividness for likelihood—judging risk not by statistics, but by how easily we can picture it. This availability-based intuition makes rare events seem common, especially when they are recent, severe, or emotionally charged. Probability neglect and base-rate neglect lead families to overestimate dramatic risks, focusing on catastrophic possibility rather than actual probability.

### Social amplification and uncertainty: When fear spreads faster than the facts

Media coverage and social media sharing create an availability cascade, reinforcing the perception that sudden cardiac death is common. Ambiguity aversion then pushes both families and clinicians toward more testing—less to improve safety, and more to reduce the discomfort of uncertainty.

### Clinician susceptibility: When experience shapes judgment

Clinicians are not immune. Personal encounters with rare or emotionally relatable cases can skew risk estimation in either direction. Fatigue, stress, and anticipated regret subtly influence judgment. Optimism and pessimism bias may lead to inconsistent counseling and screening recommendations, highlighting the need for standardized, evidence-based decision-making.

The result is a kind of emotional epidemiology—a pattern in which fear spreads more reliably than the condition itself. Recognizing these distortions matters not because families should think like statisticians, but because clinicians can anticipate how fear shapes perception and reframe risk with empathy, numbers, and context. This creates the foundation for effective counseling: not simply providing accurate risk, but reshaping how risk is understood.

## Reframing Fear: Practical Counseling Strategies

Understanding cognitive bias is valuable only if it informs how we counsel families. Drawing from both research and clinical experience, the 4 strategies outlined here are especially effective in helping families move from fear-based to informed decision-making.

### Begin with emotional validation

Fear narrows attention to worst-case scenarios, and families will not process probabilities until their emotions are acknowledged. Even a simple statement—“I understand how frightening this feels, and how upsetting it is to imagine something happening to your child”—can lower defensiveness and shift the conversation from fear to understanding.

### Make risk comprehensible using natural frequencies and meaningful comparisons

Abstract percentages rarely change perception, but simple frequencies and real-world comparisons can. For example: “Each year, over 50,000 youth athletes play organized sports. Only 1 or 2 may experience a cardiac arrest—while dozens of teenagers die driving to practice.” Families understand risk better when they can *picture* it, rather than calculate it.

### Reframe the goal: not eliminating risk, but choosing which risks to carry

Every decision carries risk—including doing nothing. Avoiding sports may feel safer, but it introduces its own risks to physical health, self-worth, peer connection, and mental well-being. The goal is not to chase zero risk—that is impossible—but to make thoughtful, values-based decisions that balance safety with the benefits of participation. A helpful pivot might be: “We can't remove all risk, but we can make wise choices that reflect what matters most for your child's health, growth, and future.”

### Use shared decision-making to restore agency and reduce anxiety

Anxiety rises when families feel powerless. Offering options—rather than prescriptions—invites them into the reasoning process: “Here's what we know, here's what remains uncertain, and here are the reasonable paths forward. Let's decide together.” Shared decision-making shifts parents from fear of regret to ownership of choice.

## Conclusions: From Fear to Perspective

The collapse of a single athlete can alter national behavior overnight, even when true risk remains minimal. To understand this response, clinicians must recognize the psychology that shapes risk perception. More importantly, we must meet these fears with empathy, connection, and context—using clear numbers, meaningful comparisons, and shared decision-making. Ultimately, the role of the cardiologist is not only to prevent tragedy, but to help families interpret risk wisely, preserve the benefits of participation, and transform fear-based decisions into informed, values-based choices.

## Funding Support and Author Disclosures

The authors have reported that they have no relationships relevant to the contents of this paper to disclose.
